# Possible Occupational Infection of Healthcare Workers with Monkeypox Virus, Brazil

**DOI:** 10.3201/eid2812.221343

**Published:** 2022-12

**Authors:** Richard Steiner Salvato, Maria Leticia Rodrigues Ikeda, Regina Bones Barcellos, Fernanda Marques Godinho, Patrícia Sesterheim, Leticia Camiza Bulcão Bitencourt, Tatiana Schäffer Gregianini, Ana Beatriz Gorini da Veiga, Fernando Rosado Spilki, Gabriel Luz Wallau

**Affiliations:** Secretaria Estadual da Saúde do Rio Grande do Sul, Porto Alegre, Rio Grande do Sul, Brazil (R. Steiner Salvato, M.L. Rodrigues Ikeda, R. Bones Barcellos, F. Marques Godinho, P. Sesterheim, T. Schäffer Gregianini);; Universidade do Vale do Rio dos Sinos Programa de Pós-Graduação em Saúde Coletiva, São Leopoldo, Rio Grande do Sul (M.L. Rodrigues Ikeda, L.C. Bulcão Bitencourt);; Universidade Federal de Ciências da Saúde de Porto Alegre, Porto Alegre, Rio Grande do Sull (A.B. Gorini da Veiga);; Universidade Feevale Laboratório de Microbiologia Molecular, Novo Hamburgo, Rio Grande do Sul (F. Rosado Spilki);; Instituto Aggeu Magalhães (IAM), FIOCRUZ-PE, Recife, Brazil (G.L. Wallau);; National Reference Center for Tropical Infectious Diseases, Hamburg, Germany (G.L. Wallau)

**Keywords:** monkeypox, fomites, healthcare personnel, patient interactions, infections, zoonoses, viruses, Brazil

## Abstract

We evaluated epidemiologic and molecular characteristics of monkeypox virus (MPXV) infections sampled from 2 healthcare nurses. Five days after collecting samples from an infected patient, the nurses showed typical MPXV manifestations; quantitative PCR and whole-genome sequencing confirmed MPXV infection, most likely transmitted through contact with fomites.

In May 2022, the World Health Organization (WHO) confirmed a multicountry monkeypox virus (MPXV) outbreak caused by MPXV clade II. As of September 14, 2022, 59,147 infections had been described in 164 countries worldwide, 6,129 of those cases occurred in Brazil (*1*). Typical MPXV signs and symptoms include fever, intense headache, lymphadenopathy, back pain, myalgia, and intense asthenia. Skin eruptions usually begin within 1–3 days after fever onset and evolve from macules to pustules, then form crusts (*1*,*2*). 

In this outbreak, most reported cases have been transmitted through sexual contact with multiple partners. However, MPXV can also be transmitted through direct contact with rash lesions, scabs, body fluids and respiratory secretions from an infected patient (*3*,*4*). Transmission through contact with fomites, infected objects, fabrics, or surfaces, has also been reported (*5*) and should be considered for disease control and prevention. By August 22, 2022, WHO had reported 256 MPXV cases among healthcare workers (HCW); only 3 of them were confirmed to be occupationally acquired. Of note, most infections among HCWs were acquired outside the workplace (*6*). 

We describe MPXV infection that developed in 2 HCWs after they collected specimens from an infected patient in Brazil. Both healthcare workers signed a consent form for the use of their clinical data and publication of anonymized photographs in this article. 

## The Study

On July 22, 2022, a man in Brazil, 40 years of age, exhibited genital maculopapular lesions, adenomegaly, myalgia, fever, and chills. The patient had not traveled recently; he reported intimate contact with multiple partners. On July 29, two HCWs (HCW-1 and HCW-2) visited the patient’s home to collect specimens and conduct an epidemiologic investigation interview. Upon entering the patient’s home and during the entire visit, the HCWs wore personal protective equipment (PPE), including safety glasses, disposable isolation gowns, and N95 respiratory masks. The patient wore a cloth mask for the duration of the visit. 

After entering the home, the patient and HCWs proceeded directly to the patient’s bedroom, where the HCWs interviewed the patient and collected samples from him. During these procedures, the patient remained in bed; the HCWs placed their equipment on a nearby armchair. From the time they entered the patient’s home to the end of the interview, the HCWs did not wear gloves; after the interview, both HCWs sanitized their hands with 70% ethanol and donned latex gloves to collect samples. HCW-1 collected a lesion specimen using a dry sterile swab that the worker then placed in a screw-capped sterile plastic transport tube; HCW-2 collected a blood sample using a plastic evacuated tube. Both tubes were stored in a sample transport box. During the ≈1 hour visit, the HCWs had no skin-to-skin contact with the patient and reported no sharps injuries. 

**Figure 1:**
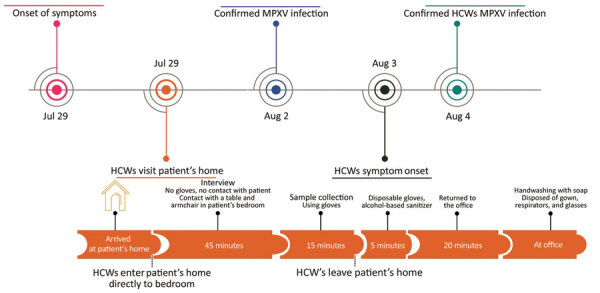
Timeline of monkeypox patient illness, HCW visit to the patient’s home, and subsequent HCW illness, Brazil, 2022. HCW, healthcare worker; MPXV, monkeypox virus.

The HCWs removed their gloves only after leaving patient’s home and placing the sample box in their car; they then discarded the gloves in a portable biohazard waste disposal container and sanitized their hands with 70% ethanol. They wore their remaining PPE (disposable gown, N95 respirators, glasses) until they arrived at the laboratory, where they immediately washed their hands with soap and water. However, they did not sanitize some work materials, such as a clipboard and the exterior surface of the sample transport box (Figure 1). 

The HCWs did not have contact with other suspected or confirmed monkeypox case-patients before the day of or during the 4 days after collecting samples from the patient. Furthermore, on the day of the patient visit, they had no known skin injuries, skin breaks, or scrapes. A real-time quantitative PCR (qPCR) assay performed on August 2 following a protocol described elsewhere (*7*) confirmed that the patient was infected with clade II MPXV (cycle threshold [Ct] 20). 

**Figure 2:**
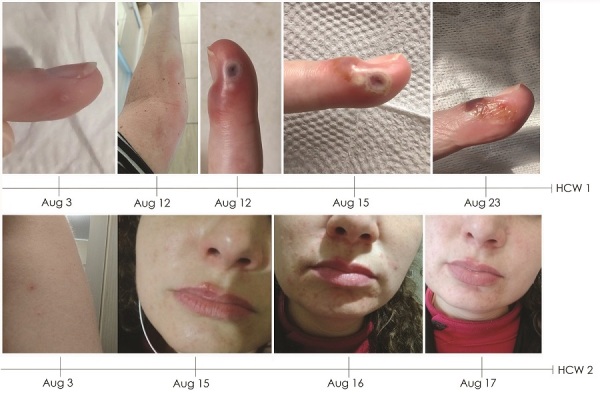
Timeline of skin lesions shown by HCW-1 and HCW-2, who had confirmed monkeypox virus infection after visit to home of monkeypox patient, Brazil, 2022. HCW, healthcare worker.

On August 3 (5 days after collecting the patient specimens), HCW-1 exhibited a single lesion on her left ring finger, a small macula with central umbilication. qPCR of a specimen collected from HCW-1 on August 4 confirmed MPXV infection (Ct 22). We observed no systemic symptoms or additional lesions until August 10, when HCW-1 experienced increased hyperemia and a small papule appeared lateral to the initial lesion. By August 12, HCW-1 exhibited lymphangitis in her left upper arm and worsened hyperemia; in addition, the lesion on her finger became a bleeding papule. On August 13, HCW-1 still had lymphangitis and a small papule had appeared on her forearm. By August 15, lesion fibrin had increased, and by August 23, fibrin reabsorption with crust formation had occurred (Figure 2). 

By August 3, HCW-2 exhibited a papule on her forearm and fever and lymphadenopathy had developed. On August 4, we confirmed MPXV infection by qPCR (Ct 36). Lesions spread to her face and increased progressively until August 16 but did not evolve to crust. The lesions began to diminish on August 17 (Figure 2) and on August 24, HCW-2 was released from isolation because all lesions had healed. 

Using the same qPCR protocol, we detected MPXV in 3 persons: the original patient, HCW-1, and HCW-2. We selected samples from the patient and HCW-1 for whole-genome sequencing because of their higher viremia. We performed whole-genome amplification as described elsewhere (*8*) and sequencing on an Illumina MiSeq sequencing platform (https://www.illumina.com), following best practices to avoid cross-contamination. We used ViralFlow (https://github.com/dezordi/ViralFlow) (*9*) for genome assembly and consensus generation, using an MPXV reference genome (GenBank accession no. MT903345.1). Analysis using the Nextclade tool (https://clades.nextstrain.org) showed that the sequenced genomes were 100% identical and belonged to MPXV clade IIb sublineage B.1.1 (Appendix Figures 1–3). We deposited consensus sequences in GISAID (accession nos. EPI_ISL_14465517 and EPI_ISL_14676265).

## Conclusions 

Our report provides evidence supporting the hypothesis that both HCW infections observed in this study were transmitted through fomite exposure with surfaces in the patient’s home, their own PPE, or outer surfaces of the specimen transport box. These findings highlight that MPXV might be acquired through contact with fomites, such as patient belongings or surfaces contaminated with infectious viral particles. Close interaction between patients and HCWs are also risk factors for MPXV transmission (*4*). As notable routes of MPXV transmission, such interactions should be targeted along with diagnosis and quarantine for MPXV containment measures (*4*). Recommendations for preexposure and postexposure prophylaxis include correct use of appropriate PPE (*10*,*11*). Infectious MPXV particles can remain on furniture and fabric surfaces (*12*), so caution is needed when in contact with general protection equipment and household objects that have been exposed to suspected case-patients. 

We propose specific measures to prevent and curtail monkeypox infection acquired through fomites. HCWs must be properly trained to safely collect specimens, use PPE, implement control measures, and perform frequent hand hygiene. HCWs should wear gloves throughout entire visits and during contact with possibly infected persons and their belongings. Secondly, a disinfectant product effective against microbial pathogens such as nonenveloped viruses (e.g., norovirus, rotavirus, adenovirus, poliovirus) should be applied to object surfaces before and after interactions with suspected case-patients. Finally, vaccination campaigns should be conducted among high-risk groups, including certain HCWs. The possible transmission of MPXV by 2 HCWs from a patient environment illustrates a potential source of transmission with broad implications for infection control and prevention and indicates the need for specific interventions in the context of the ongoing multicountry outbreak. 

Appendix Additional information on rfeport of occupationally acquired monkeypox in 2 healthcare nurses.

## References

[1] World Health Organization. WHO health emergency dashboard [cited 2022 Aug 26]. https://extranet.who.int/publicemergency

[2] World Health Organization. Monkeypox [cited 2022 Aug 26]. https://www.who.int/news-room/fact-sheets/detail/monkeypox

[3] McCollum AM, Damon IK. Human monkeypox. Clin Infect Dis. 2014;58:260–7. 24158414 10.1093/cid/cit703 PMC5895105

[4] Vaughan A, Aarons E, Astbury J, Brooks T, Chand M, Flegg P, et al. Human-to-human transmission of monkeypox virus, United Kingdom, October 2018. 10.3201/eid2604.191164 PMC7101111 32023204

[5] Mauldin MR, McCollum AM, Nakazawa YJ, Mandra A, Whitehouse ER, Davidson W, et al. Exportation of monkeypox virus from the African continent. J Infect Dis. 2022;225:1367–76. 32880628 10.1093/infdis/jiaa559 PMC9016419

[6] World Health Organization. Multi-country outbreak of monkeypox, External situation report #4—24 August 2022 [cited 2022 Aug 26]. https://www.who.int/publications/m/item/multi-country-outbreak-of-monkeypox--external-situation-report--4---24-august-2022

[7] Li Y, Zhao H, Wilkins K, Hughes C, Damon IK. Real-time PCR assays for the specific detection of monkeypox virus West African and Congo Basin strain DNA. J Virol Methods. 2010;169:223–7. 20643162 10.1016/j.jviromet.2010.07.012 PMC9628942

[8] Chen NFG, Gagne L, Doucette M, Smole S, Buzby E, Hall J, et al. Monkeypox virus multiplexed PCR amplicon sequencing (PrimalSeq) V.2. V.2 [cited 2022 Aug 26]. https://www.protocols.io/view/monkeypox-virus-multiplexed-pcr-amplicon-sequencin-cd8ds9s6

[9] Dezordi FZ, Neto AMDS, Campos TL, Jeronimo PMC, Aksenen CF, Almeida SP, et al.; On Behalf Of The Fiocruz Covid-Genomic Surveillance Network. ViralFlow: a versatile automated workflow for SARS-CoV-2 genome assembly, lineage assignment, mutations and intrahost variant detection. Viruses. 2022;14:217. 35215811 10.3390/v14020217 PMC8877152

[10] Rao AK, Petersen BW, Whitehill F, Razeq JH, Isaacs SN, Merchlinsky MJ, et al. Use of JYNNEOS (smallpox and monkeypox vaccine, live, nonreplicating) for preexposure vaccination of persons at risk for occupational exposure to orthopoxviruses: recommendations of the Advisory Committee on Immunization Practices—United States, 2022. MMWR Morb Mortal Wkly Rep. 2022;71:734–42. 35653347 10.15585/mmwr.mm7122e1 PMC9169520

[11] World Health Organization. Vaccines and immunization for monkeypox: interim guidance, 24 August 2022 [cited 2022 Aug 26]. https://www.who.int/publications/i/item/WHO-MPX-Immunization-2022.2-eng

[12] Morgan CN, Whitehill F, Doty JB, Schulte J, Matheny A, Stringer J, et al. Environmental persistence of monkeypox virus on surfaces in household of person with travel-associated infection, Dallas, Texas, USA, 2021. Emerg Infect Dis. 2022;28:1982–9. 35951009 10.3201/eid2810.221047 PMC9514334

