# Supplementary material for: Possible Occupational Infection of Healthcare Workers with Monkeypox Virus, Brazil
Source: Emerg Infect Dis. 2022 Dec;28(12):2520–3. doi: 10.3201/eid2812.221343 (PMC9707598; doi:10.3201/eid2812.221343)
Supplement: Appendix — Additional information on rfeport of occupationally acquired monkeypox in 2 healthcare nurses. [file 22-1343-Techapp-s1.pdf]

Article DOI: https://doi.org/10.3201/eid2812.221343

Possible Occupational Infection of Healthcare Workers with Monkeypox Virus, Brazil

Appendix

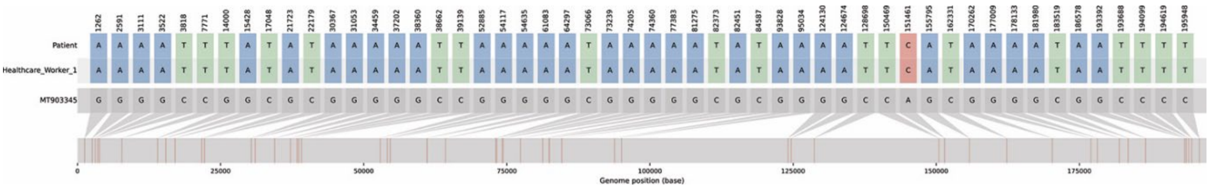

Appendix Figure 1. Single nucleotide polymorphisms of the MPXV genomes from patient and HCW-1 with respect to the reference genome. Figure was rendered using the Snipit tool (https://github.com/aineniamh/snipit).

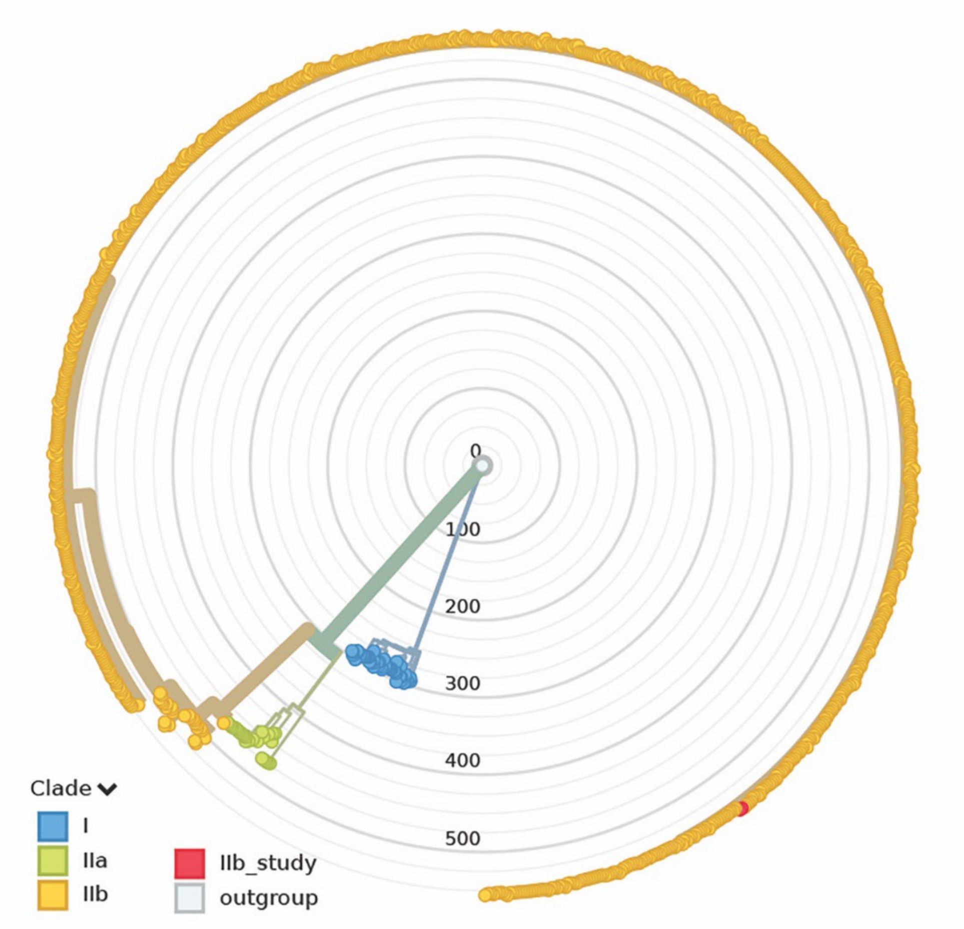

Appendix Figure 2. Maximum likelihood phylogenetic tree showing the 2 sequenced genomes in this study (red circles) along with the worldwide Nextclade subsampled dataset of 919 MPXV sequences.

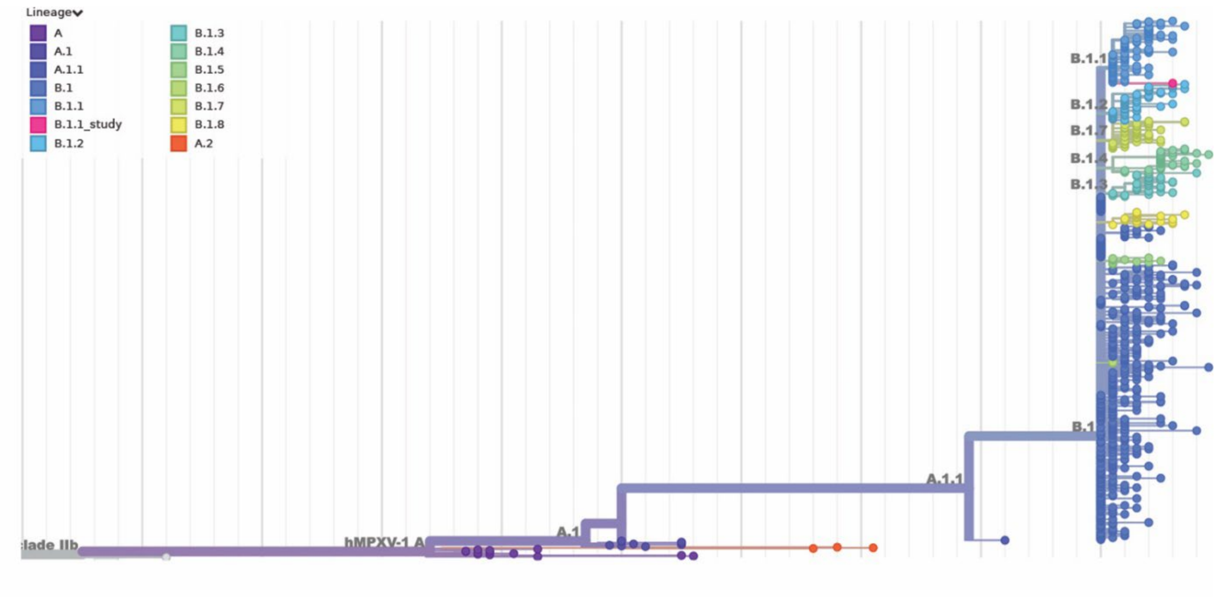

Appendix Figure 3. Phylogenetic tree focusing on the MPXV Clade IIb.
